# OGRE: calculate, visualize, and analyze overlap between genomic input regions and public annotations

**DOI:** 10.1186/s12859-023-05422-w

**Published:** 2023-07-26

**Authors:** Sven Berres, Jörg Gromoll, Marius Wöste, Sarah Sandmann, Sandra Laurentino

**Affiliations:** 1 Centre of Reproductive Medicine and Andrology, University of Münster, Albert-Schweitzer-Campus 1 Building D11, 48149 Munster, Germany; 2 Institute of Medical Informatics, University of Münster, Albert-Schweitzer-Campus 1 Building A11, 48149 Munster, Germany

**Keywords:** Annotation, Genomic association, Genomic regions, Omics, Overlap, Regulatory elements, Shiny, Visualization

## Abstract

**Background:**

Modern genome sequencing leads to an ever-growing collection of genomic annotations. Combining these elements with a set of input regions (e.g. genes) would yield new insights in genomic associations, such as those involved in gene regulation. The required data are scattered across different databases making a manual approach tiresome, unpractical, and prone to error. Semi-automatic approaches require programming skills in data parsing, processing, overlap calculation, and visualization, which most biomedical researchers lack. Our aim was to develop an automated tool providing all necessary algorithms, benefiting both bioinformaticians and researchers without bioinformatic training.

**Results:**

We developed overlapping annotated genomic regions (OGRE) as a comprehensive tool to associate and visualize input regions with genomic annotations. It does so by parsing regions of interest, mining publicly available annotations, and calculating possible overlaps between them. The user can thus identify location, type, and number of associated regulatory elements. Results are presented as easy to understand visualizations and result tables. We applied OGRE to recent studies and could show high reproducibility and potential new insights. To demonstrate OGRE’s performance in terms of running time and output, we have conducted a benchmark and compared its features with similar tools.

**Conclusions:**

OGRE’s functions and built-in annotations can be applied as a downstream overlap association step, which is compatible with most genomic sequencing outputs, and can thus enrich pre-existing analyses pipelines. Compared to similar tools, OGRE shows competitive performance, offers additional features, and has been successfully applied to two recent studies. Overall, OGRE addresses the lack of tools for automatic analysis, local genomic overlap calculation, and visualization by providing an easy to use, end-to-end solution for both biologists and computational scientists.

**Supplementary Information:**

The online version contains supplementary material available at 10.1186/s12859-023-05422-w.

## Background

Modern genome sequencing produces ever-growing numbers of large genomic datasets for multiple organisms. Consequently, processing and putting these data into biologically meaningful context remains a challenge. Databases like Ensembl and UCSC store this information and list an increasing amount of annotation data [1, 2]. In the human genome, there are now over 20,000 known protein-coding, 40,000 micro (miRNA), and 19,000 long non-coding (lncRNA) RNA genes, 252,000 transcripts, 30 million CpG sites, and 5 million single nucleotide polymorphisms (SNPs) [3, 4]. Apart from this, the amount of available data on the location of epigenetic markers—such CpG islands (CGI), histone modifications, and chromatin 3D structure—and regulatory regions—e.g. promoters and enhancers—has also grown in recent years [5, 6]. These genomic elements can occupy regions from a few base pairs up to several mega base pairs and are not randomly distributed throughout the genome. Regions that are overlapping or neighboring each other might interplay and perform potential regulatory functions. A prime example are promoter regions, typically located upstream from the transcription start site (TSS) [7] and involved in the regulation of gene expression (8, 9), which often include transcription factor binding sites (TFBS) and CGIs.

Techniques such as RNA sequencing, methylome analysis, chromatin immunoprecipitation (ChIP) sequencing, and whole genome association studies often result in a set of candidate genes or a collection of interesting genomic regions, which need to be further investigated by researchers who are not always trained in using bioinformatic and data processing tools. Especially, the validation and further downstream analysis of candidate genes resulting from differential gene expression analysis benefits from information about the various regulatory elements controlling gene transcription. This requires acquiring, mining, and parsing multiple datasets for the overlap of regions with e.g. promoters, TFBS, CGIs, and other regulatory elements. A manual approach to identify TFBS and CGIs within a gene’s promoter region, for instance, is not trivial. The first step—to mine published annotations for overlaps with a list of candidate genes—requires obtaining genomic coordinates for each candidate and manually searching for the features of interest in one of the various genome browsers (e.g. UCSC or Ensembl). One would then need to make sure to obtain annotations for all regulatory elements of interest and visualize them using appropriate software (e.g. IGV browser), which might not be easily achievable. In the next step, overlapping or neighboring regulatory elements need to be identified and annotated. Finally, genomic locations, results, and graphics must be ponderously exported for further processing. This manual approach can be tiresome, unreliable, and prone to errors, especially for long candidate lists. It further leads to non-comparability and varying results depending on the person conducting the manual analysis. In addition, most researchers might not have the bioinformatics expertise necessary for retrieving and visualizing the results. Nevertheless, this kind of analysis remains a key element in understanding the interplay between regulatory elements and thus the observed gene expression changes under different experimental conditions. Therefore, a tool allowing researchers to identify the presence of regulatory elements for a set of genomic regions in a user friendly and platform-independent way is urgently needed. To our knowledge, no software tool is available that allows the automation of these tasks, and such a complex analysis still requires the help of a bioinformatician or computational scientist to do the necessary data parsing and programming. Therefore, we developed OGRE (Overlapping annotated Genomic Regions) as a user friendly and easily accessible tool to perform automatic overlap analysis, export tabular results, and visualize genomic regions based on publicly available annotations. In addition, the user interface SHREC (SHiny interface for REgion Comparison) provides accessibility for biologists without computational training.

## Implementation

### Workflow

**Fig. 1:**
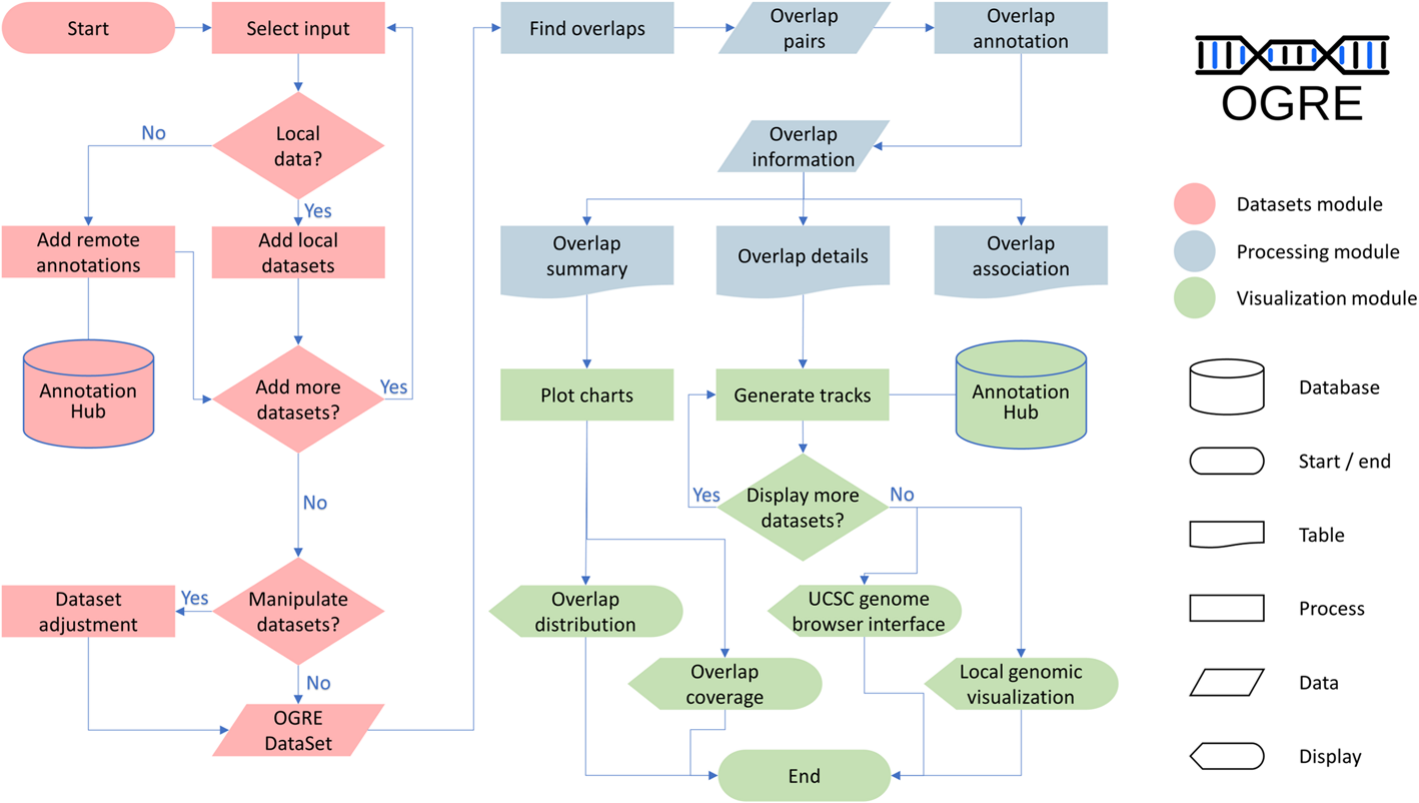
OGRE workflow. OGRE’s architecture is divided into three modules: Datasets (red), Processing (blue), and Visualization (green) Database access is interconnected with key processes, data generation, results generation, and visualization. Decision junctions (rhombus shaped) display the user’s options to influence number and type of datasets, dataset manipulation and visualization parameters

Internally OGRE methods are structured in three modules listed as follows: (1) Dataset module, (2) Processing module, and (3) Visualization module (Fig. 1). We further define an OGREDataSet as a list of datasets with additional metadata information that serves as input for each module. The Dataset module reads user-generated local tabular data like .CSV and .GFF files which often result from OMICS experiments. Once the user defines a directory, it is scanned for suitable file types, which are attached to the OGREDataSet, enabling read-in of multiple datasets at once. External datasets show a wide range of file formats, structures, format, and naming conventions and are therefore not immediately ready for an overlap analysis. OGRE offers a growing number of built-in annotations for promoters, genes, CpG islands, SNPs, and TFBS. This is achieved by parsing functions that scan those datasets for duplicates, chromosome naming conventions, genome build and version differences. In addition, we provide instructions on how to process datasets from different origins. As illustrated in Fig. 1, the user is able to add and modify datasets within the Dataset module at any point. Integrated convenience functions allow resizing of input elements, making it possible to focus on specific regulatory regions like promoters or other up/downstream areas. For instance, dataset coordinates can be modified relative to the start/end positions, taking the DNA strand information into account (e.g. (−) 1200 bp from TSS). Next, overlap calculation is started by the Processing module, which operates on any supplied OGREDataSet and can be adjusted for multiple parameters like the minimum overlap required for two regions, type of overlap (i.e. full or partial), and strand-specific overlaps. The resulting hits, a pair of overlapping regions, is then further annotated by extracting genomic coordinates for each involved region pair, and used to generate tables containing comprehensive information underlying each overlap. In detail, the table contains genomic coordinates for both region pairs and for the overlapping region itself, length of overlap, and reports the overlapped nucleotide fraction with respect to the original input region. Some regions exhibit low overlap numbers whereby others, for example in promoter-TFBS or intergenic regions-SNP associations, typically show multiple overlaps. OGRE offers routines for extracting all elements overlapping a single region and thus identifies regions with many or few overlaps. Some genomic elements cluster around regulatory regions such as TFBSs upstream of genes. We therefore expect distinct coverage profiles, caused by an overlap enrichment at certain areas. To measure this, we divide all regions of a dataset of interest into 100 equally sized bins. In a next step we sum up all elements of a second dataset that fall into each of the bins. For a genes-TFBS dataset, this means every gene body is split into 100 bins, whereby the first bins start with the gene transcription start site and the last bins end with the gene transcription termination site. A matrix stores this information for all first dataset’s regions and a vector is defined containing the accumulated overlap coverage along the bins. A summary table displays informative statistics such as minimum, lower quantile, mean, median, upper quantile, and maximum number of overlaps per region and per dataset. The last module, Visualization, illustrates the summary table’s information as bar plots and generates histograms to display overlap distributions by grouping the number of overlaps into predefined bins. Chromosome, strand, start, and end coordinates of all datasets are then used to generate tracks for a local genomic visualization representing a user-defined genome window. Optionally, multiple layers of datasets, that were not directly part of the overlap calculation, can be displayed alongside the initially selected datasets. Appearance like colors, shapes, and labeling types can be adjusted and taken into account by the user. As an alternative exploration method, we implemented an interface to display overlapping regions on public genome browsers.

**Fig. 2:**
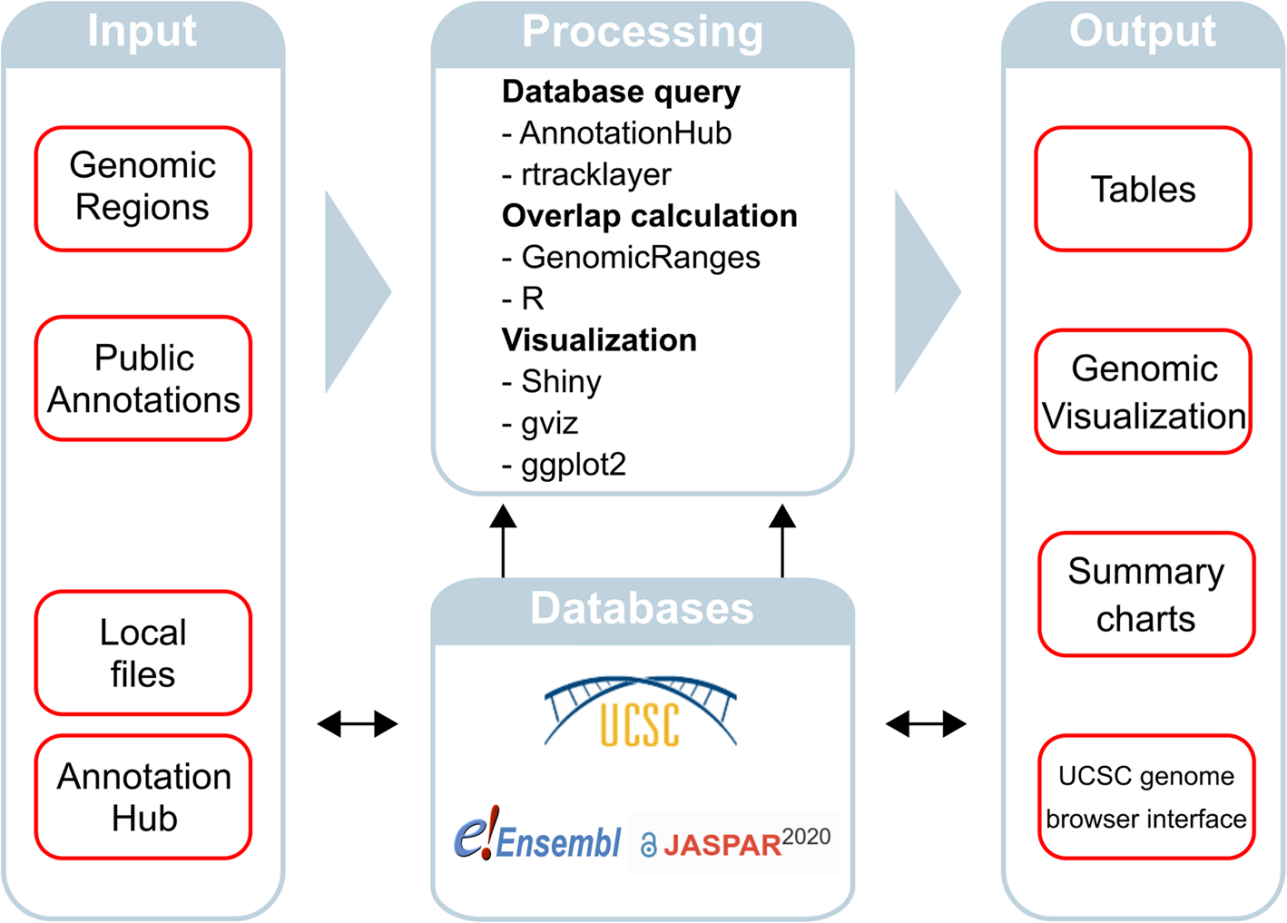
Graphical representation of OGRE's functionality. Input of genomic regions of interest and public annotations by reading in local files or connecting to AnnotationHub. Input data is processed and results are presented as output in the form of tables, genomic visualization, charts, and a UCSC genome browser interface

On the technical side, OGRE was programmed in R 4.1.0 [10] using the RStudio integrated development environment [11] and visualization is done with Shiny [12]. OGRE’s structure is displayed in Fig. 2, where input, processing, and output are interconnected with annotations from public databases. Most functionalities were implemented with the R base code and the use of additional packages, namely GenomicRanges [13] to calculate overlap between input regions and public annotations, DataTable [14] for efficient data storage, AnnotationHub to obtain public annotations [15], Gviz [16] and ggplot2 [17] for result visualization and region plotting in genomic space, and shinyBS [18] for user tooltips. OGRE is available on Biocoductor (https://bioconductor.org/packages/devel/bioc/html/OGRE.html), GitHub (https://github.com/svenbioinf/OGRE), and we developed SHREC (SHiny interface for REgion Comparison) as a user-friendly interface from which OGRE can be accessed under any operating system’s default internet browser using R. The tool’s vignette offers users an example run and a frequently asked question section. On GitHub, we provide additional documentation, installation options (Docker, GitHub), and a tutorial video on how to use OGRE and its graphical user interface.

*Input* With OGRE, one or multiple genomic regions (e.g. genes) can be supplied to the input text field, selected from built-in annotations, or alternatively supplied as tabular files containing genomic regions. The tool is compatible with regions in BED or GFF formats, such as those obtained from ChIP-Seq, methylome sequencing, and public annotations with chromosome, start, and end information. Next, several regulatory elements must be selected or, alternatively, uploaded by the user as a tabular file with genomic ranges. By default, OGRE offers promoter (Ensembl) [19], CpG islands (UCSC) [20], and TFBS [21] annotations. Different parameters can be set to modify already uploaded regions by extending their start/end positions, focus on regions of interest (e.g. promoter regions of genes), or subset the data (e.g. focus on cell-specific TFBS).

**Fig. 3:**
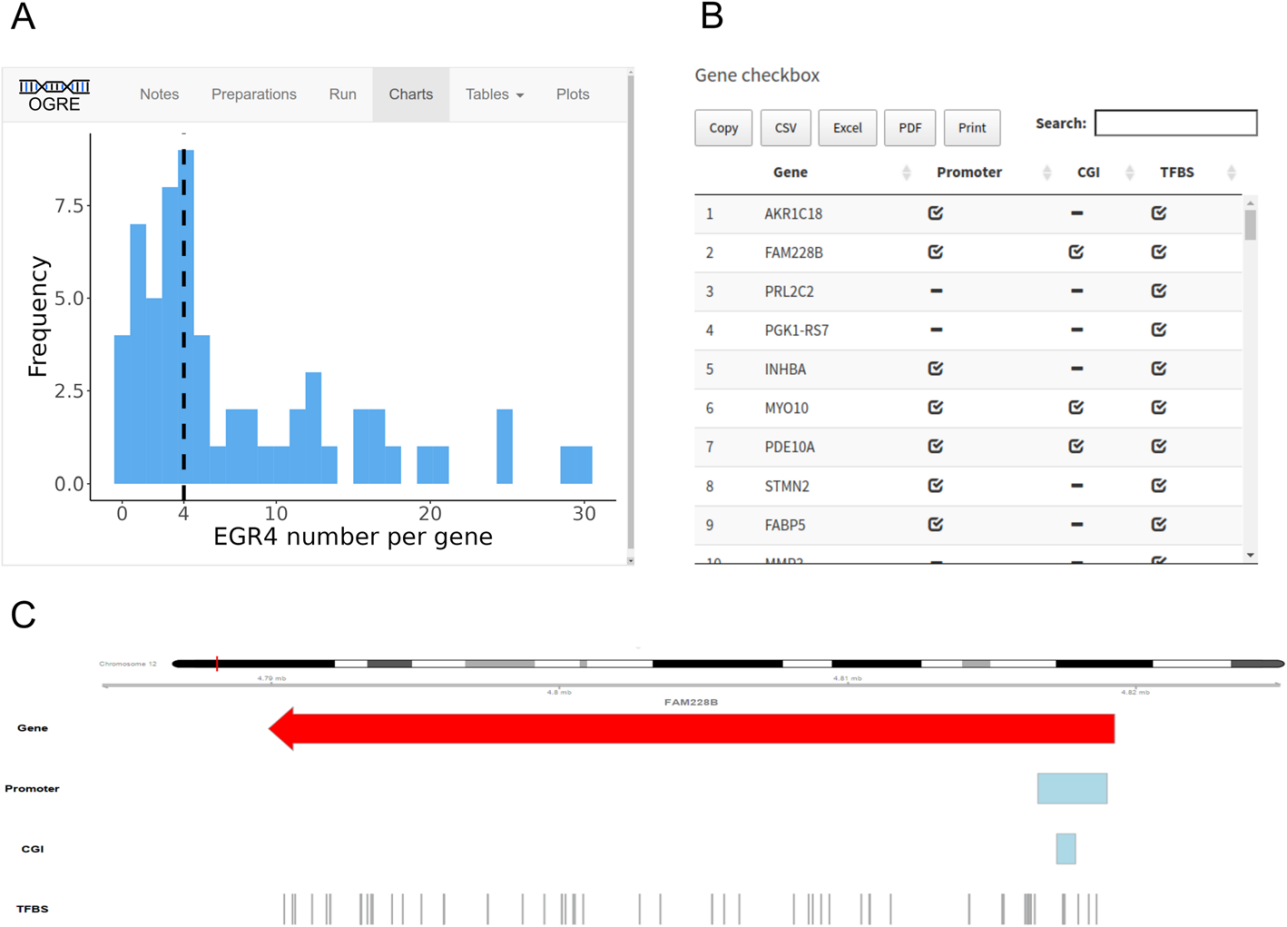
Application of OGRE for a list of genes following a differential gene expression experiment and display of user interface SHREC. **A** OGRE’s graphical user interface with a histogram chart displaying a distribution of EGR4 binding sites with median as dashed black line. Y-axis: EGR4 binding site frequency, x-axis: Number of EGR4 binding sites per gene. **B** Gene checkbox listing regulatory element presence; promoter, CGI, and TFBS in a set of input genes. **C** Genomic view window of FAM228B with strand information and promoter, CGI and TFBS without strand information

*Processing* Depending on input data and settings, the tool extracts genomic coordinates from user-supplied internal (by reading local files) or external data (by connecting to public databases). User-supplied tabular files are parsed and stored as GenomicRanges objects for easy access during the session. External datasets are available on the AnnotationHub web resource, which hosts genomic annotations from various sources. OGRE can access these files through an internal interface and provides a number of default regulatory element annotations already optimized for analysis. Like this, annotations are packaged separately and independent from the actual software tool. Parsing and filtering operations ensure homogeneous naming and structure schemes between the different annotation files. Once all information is available, overlaps between the input regions and all selected annotations are computed, whereby both complete and partial overlaps are considered. For this analysis, OGRE as well as other similar tools uses the efficient findOverlaps() implementation of the GenomicRanges package to conduct pairwise comparisons between query (input regions) and subject (public annotations). A hit describes the overlap of query and subject in at least one nucleotide base. Chromosome, start, end position, and overlap (in percentage and number of base pairs overlapping) of each hit are stored in a data table for downstream analysis (Additional file 5: Table S2, Additional file 6: Table S3). In addition, OGRE reports the number of query regions, calculates the total number of annotation types found among query regions, regions with at least one regulatory element, and the average number of regulatory elements per query. Results are internally stored as data tables, which can be exported (Fig. 3B, Additional file 5: Table S2, Additional file 6: Table S3) and are in turn the input for visualization with ggplot2 and Gviz (Fig. 3A, C). Shiny is used to set up the convenient user interface SHREC. In more detail, we visualize input and output data with the ggplot2 [17] R package to create basic bar plots with information on the number of submitted queries/genes. Furthermore, the total and average number of subjects/regulatory elements found for every input is computed. OGRE makes extensive use of the DT [14] R package to display results as HTML tables, which can also be set to display detailed information for single elements. Those tables are integrated within OGRE’s user interface, can be exported in a variety of file formats like .CSV and .PDF, and offer interactive filtering capability (Fig. 3B). Each query element (e.g. gene) can be explored in detail using the UCSC genome browser interface [22] or OGRE’s genomic visualization feature, which displays the region of interest and additional user-defined genome tracks (Fig. 3C).

*Output* Results can be downloaded or accessed as various table formats like .CSV and .PDF files and graphical representations like PNG and JPEG files. Long and wide format tables provide different levels of information whereby the former contains comprehensive information of all regions with additional details and the latter offers a quick overview of the regions with all associated regulatory features in one line. Metrics (e.g. average/min/max number of overlaps) are displayed within a summary chart and the query/gene checkbox provides compact information on the overlap distribution, which can also be individually explored in the genomic visualization feature. Once a query/gene is selected the genomic coordinate information is used to create a static local image focused on the query region, showing all subject/regulatory elements with multiple adjustable parameters for an optimal visualization. The same information is sent to the UCSC genome browser interface, as an external exploration tool.

## Results

In order to test and show the OGRE’s application to real biological data, we used the tool on two studies with publicly available datasets, which were originally analyzed using different methods.

### Transcription factor binding sites within differentially expressed genes’ promoters

**Fig. 4:**
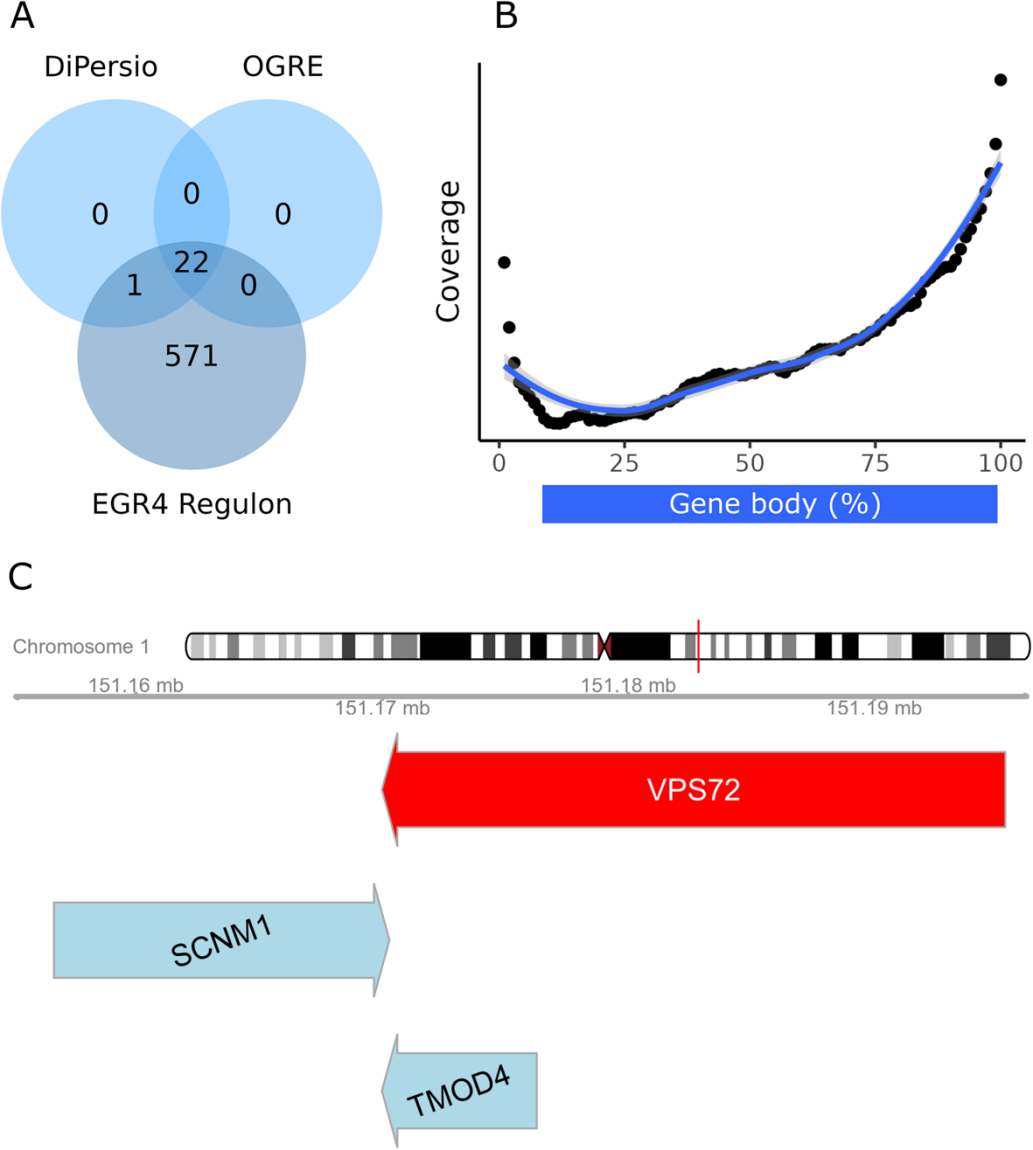
Analysis output **A** Overlap between genes analyzed by Di Persio et al. [23] and OGRE. Di Persio and Tekath et al*.* identified 23 genes regulated by EGR4. OGRE identified 22 of the 23 genes and provides EGR4 binding site information. **B** Average coverage profile of all genes-gene overlaps, split in 100 bins, which represent gene bodies of all 5407 genes. **C** Overlapping genes. Three representative genes (*VPS72*, *SCNM1*, *TMOD4*) with complete (*VPS72*, *TMOD4*) and partial overlap (*VPS72*, *SCNM1*)

In a recent in-house study, Di Persio et al. [23] performed single-cell RNA sequencing on human germ cells with normal and impaired spermatogenesis. As a result, 61 genes were uniquely differentially expressed for undifferentiated spermatogonia between the two conditions. In an analysis using SCENIC [24], 23 of those genes were included in the EGR4 regulon. Therefore, the authors hypothesized that EGR4 could serve as a potential spermatogonia regulator. To evaluate if EGR4 TFBSs could be found in the same proportion of genes using OGRE, we loaded the list of differentially expressed genes (DEGs) as input. Following original sequencing parameters, we used GENCODE v30 release gene annotations [25] and TFBS information from the built-in JASPAR annotations, filtered for EGR4. Any potential overlap (partial and complete overlap and ignoring strand information) between DEGs and EGR4 binding sites was considered a hit. After a successful run, the tool provided location information and distribution of hits. Interestingly, the DEGs show a high presence of possible EGR4 TFBSs resulting from the calculated gene-TFBS overlap. In fact, 57 DEGs (93%) contain at least one EGR4 binding site with a median of 4 EGR4 binding sites per gene (min = 0, max = 30). The gene with most binding sites for EGR4 is *ST3GAL4*, whereby no overlaps could be found for genes *C1QTNF12*, *ENHO*, *MAGEB2*, and *RPL36A*. The same analysis was carried out for all GENCODE v30 release genes resulting in a mean of 3 EGR4 binding sites per gene (min = 0, max = 439), which, when compared to DEGs, is significant (*p* value ≤ 0.05) using a Wilcoxon rank-sum test (Additional file 3: Fig. S2). In addition, when we compared the list of OGRE’s output genes with EGR4 regulated genes reported by Di Persio and Tekath et al., we could identify all genes except for one (*ENHO*; Fig. 4).

### Overlapping protein-coding genes in the human genome

Overlapping genes are defined as two or more genes sharing the same location by partially or entirely overlapping with each other. They exist mostly in compact genomes like those of virus and bacteria, however they are also found in the human genome. Their close genomic proximity results in sharing the same chromatin domains or compartments, which in turn leads to parallel regulation and transcription [26]. In a recent study Chen et al. [27] analyzed 19,200 well-annotated protein-coding genes and determined that 4951 (26%) of those overlapped with adjacent genes, with the biggest cluster containing 22 overlapping genes. In an effort to match the original analysis parameters, we used Ensembl’s GRCh38.p12 gene annotation release from April 2018 and filtered the dataset for protein-coding genes with description only. After running OGRE with this similar dataset of 19,308 protein coding genes, we report a total of 5407 (28%) genes overlapping with at least one other gene. These are 456 genes, 2% more than those identified by the authors. Both partial and complete overlaps were considered as hits, reported independently from DNA strand notation (i.e. forward and reverse), and were displayed using OGRE’s local visualization feature (Fig. 4C). On average, OGRE reported 0.3 overlaps per gene (min = 0, mean = 0.3, max = 22), with most overlaps found within the protocadherin gamma family cluster. Gene–gene overlaps tend to occur more often around gene start (5′) and end (3′), whereby overlaps around the center of the gene are less frequent (Fig. 4B).

### Comparison to other tools

An overlap analysis between the user-defined regions and selected genomic annotations should be user-friendly, comprehensive, fully automated, be able to process multiple regions at once, provide annotation and detection for common regulatory elements e.g. CGI, TFBS, and promoters, and have the options to visualize and export results. The research community already offers a range of different algorithms and tools to predict or annotate genomic regions. We selected available tools with comparable features to OGRE and listed their performance among the different requirement categories (Table 1). Most tools are specialized on analyzing regions for a certain type of annotation and do not offer support for additional annotations. For example, INSECT, CiiiDER, and ConTra v3 feature prediction of TFBSs from position frequency matrices (PFM), iProEP focusses on the prediction of promoters and GaussianCpG on CGI identification. While these tools try to annotate regions based on predictions, Goldmine, regioneR, annotatr, and OGRE make use of already published annotations. We have benchmarked these packages for their overlap performance using microbenchmark [28], resulting in comparable runtimes (Goldmine 0.046 s, regioneR 0.040 s, annotatr 0.049 s, and OGRE 0.047 s) using identical input datasets, when calculating gene–gene overlap (Additional file 4: Table S1, Additional file 2: Fig. S1 and Additional file 1). All four tools report a total overlap of n = 10,014 by processing a dataset with 20,314 genes. regioneR and annotatr focus on the statistical analysis of genomic regions and do not offer a graphical user interface and genomic overlap plotting. OGRE on the other hand, excels by providing built-in annotations, processing of multiple input regions, and visualization of overlap at a genomic level, accessible through a convenient user interface (Fig. 3A).

**Table 1:** Feature comparison between OGRE and eight similar tools

Tool	Multiple input regions	Included annotations			Local genomic visualization
		CGI	TFBS	Promoter	
INSECT [29]	✓	✕	✓	✕	✓
Ciiider [30]	✓	✕	✓	✕	✓
ConTra v3 [31]	✕	✕	✓	✕	✓
regioneR [32]	✓	✕	✕	✕	✕
iProEP [33]	✓	✕	✕	✓	✕
annotatr [34]	✓	✓	✕	✕	✕
Goldmine [35]	✓	✕	✕	✕	✕
GaussianCpG [36]	✓	✓	✕	✕	✕
OGRE	✓	✓	✓	✓	✓

Software tools with similar features were compared to OGRE on their capability to manage multiple input regions, built-in annotations, and visualize overlaps.

## Discussion

OGRE was developed as a free and user-friendly tool to associate, overlap, and visualize a list of genomic input regions with publicly available annotations, which are stored in databases or are produced by specialized software packages [37–39]. OGRE is compatible with their output and can therefore enrich already existing analysis pipelines. It can handle multiple annotations with thousands of genomic elements and shows a high degree of automation, while at the same time ensuring reproducible analysis steps and result outputs. It is easy to install and useable by scientists without computational training, through the use of an intuitive user interface. A custom file upload function provides maximum input flexibility for various types of genomic regions. In addition, it contains built-in annotations, which can also be expanded in the future to include further regulatory elements or regions. Convenience functions enable detailed and summary tables, helpful charts, and a local genomic visualization and coverage option (Fig. 3A, C).

### Comparison to other tools

Our software package meets the existing need for an easy-to-use tool for the analysis and visualization of input regions and their overlap with genomic annotations. While tools to predict or use public annotations for a set of input regions exist, most of them lack essential features required for a comprehensive software solution. Not all packages are able to process multiple input regions at once or support different types of annotation and have them immediately ready for use. This is especially relevant considering the number of input regions that modern omics experiments yield. A sequential manual search for every query is not practical for large datasets. Built-in datasets (e.g. protein coding genes, promoters, single nucleotide polymorphisms, CpG-islands) present valuable shortcuts to the otherwise tedious manual download and data parsing steps that often hinder analysis progress for users without computational training. Especially for promoter regions, where most other tools or manual approaches define promoters as an arbitrary number of nucleotides upstream/downstream of TSS, we offer a built-in alternative. OGRE provides promoter annotations taken from Ensembl’s regulatory build, which is based on computationally and experimentally derived TFBS. Apart from the tools in Table 1, overlap analysis such as that performed by OGRE is carried out on an individual basis with variable quality and reproducibility. This is a direct result of scientists’ varying degrees of computational skills. With OGRE, we provide a reproducible workflow for both bioinformaticians and scientists without computational training, with a convenient user interface, lacking in most other packages. The annotation-based tools regioneR, Goldmine, annotatr, and OGRE, are all based on the GenomicRanges package and therefore show a comparable runtime for overlap calculation and mainly differ in usability, scope of application and available features. They all report the same number of overlaps, since they share the same overlap calculation implementation. With this in mind, the focus of OGRE lies on ease of use, built-in annotations, coverage analysis, and local genomic visualization options of the calculated overlap.

### Application to published datasets

To demonstrate OGRE’s use in everyday research and its ability to contribute to real research questions, we applied the tool to data from two recent studies. The first study demonstrates the tool’s potential as a valuable option/addition for downstream analysis. The authors identified EGR4 as a potential gatekeeper regulating the change in transcriptional profiles in spermatogonia [23]. We used OGRE to report and summarize any possible overlaps between the DEGs and EGR4 binding sites. The TFBSs for EGR4 were present in 93% of DEGs with a median of 4 TFBSs per gene and present in 62% of GENCODE v30 release genes with a median of 3 TFBS per gene, suggesting a potential EGR4 regulation of those DEGs genes. In addition, we were able to match 22 of the 23 EGR4 regulated genes previously identified by the authors, indicating OGRE’s good overall reproduction capabilities. One gene (*ENHO*), was not listed by OGRE, since all nearby TFBS did not pass the confidence threshold (*p* value ≤ 10^−4^) given by JASPAR’s default TBFS annotation [21]. To alter this behavior, OGRE supports additional annotations with alternative thresholds. Moreover, utilizing OGRE’s local genomic visualization distribution, the density of EGR4 binding sites can be assessed and displayed. Reported genomic coordinates describing where exactly gene-TFBS overlaps take place, can be the basis of follow-up experiments for those genes.

In the second study evaluated, Chen et al. [27] demonstrated the extent of overlapping protein-coding genes. Here, OGRE was capable of generating similar results to an already studied question. Using a similar dataset of 19,308 protein-coding genes as input regions, we report a total of 5407 genes overlapping with at least one other gene. The 2% observed difference can be accounted to varying input parameters, e.g. genome versions and gene/chromosome filtering steps. Nevertheless, using OGRE we were able to reproduce the number of reported overlapping protein-coding genes. The tool’s overlap coverage feature shows that genes tend to overlap preferably at the gene start and gene end and less often within or around the gene body center. It is also possible to monitor overlap coverage for forward and reverse oriented genes. Coverage profiles generated in this way provide new insights on distribution of genomic elements around regions of interest. Visualization on a local scale enables the user to better understand the composition and location of all involved elements, as shown in Fig. 3.

## Conclusions

Overall, OGRE can be applied to a variety of datasets in the field of genomics and is especially suited for finding overlapping public annotations for a set of input regions that the user is then able to further display and study, using the tool’s rich analysis and visualization features. As demonstrated by analyzing two publicly available datasets and comparing OGRE to similar software, we could show a competitive performance and additional integrated functions in a direct comparison. Particularly, researchers without computational training benefit from the tool’s flexibility, ease of use, and intuitive interface to produce standardized results from a reproducible workflow.

## Availability and requirements

Project name: OGRE.

Project home page: OGRE is available as Bioconductor package with integrated user interface SHREC (https://bioconductor.org/packages/devel/bioc/html/OGRE.html). On GitHub (https://github.com/svenbioinf/OGRE) tutorials and alternative installation options (Docker, GitHub) are provided.

Operating system(s): Platform independent.

Programming language: R.

Other requirements: Bioconductor.

License: Artistic-2.0.

Any restrictions to use by non-academics: None.

## Supplementary Information

**Additional file 1.** Benchmark script. R code used for benchmarking four overlap tools in terms of processing time and number of overlaps.

**Additional file 2**: Figure S1. Computation times. Benchmark of overlap calculation by tools Goldmine, regioneR, annotatr and OGRE using two, 20,000 lines long input files with 10 runs each, computation time reported in milliseconds.

**Additional file 3**: Figure S2. EGR4 TFBS. Number of EGR4 TFBS of all genes when comparing DEGs and GENCODE v30 release genes.

**Additional file 4**: Table S1. Benchmark statistics. Benchmark statistics of overlap calculation by tools Goldmine, regioneR, annotatr, and OGRE showing detailed calculation times (min, lq, mean, median, uq, max).

**Additional file 5**: Table S2. OGRE tabular output 1. OGRE’s tabular output of gene- TFBS overlap, where each gene is listed with all overlapping TFBS using input data from [23].

**Additional file 6**: Table S3. OGRE tabular output 2. Detailed information on each occurring overlap including genomic coordinates for TFBS using input data from [23].

## Data Availability

The datasets supporting the conclusions of this article are included within the article and its additional files. In addition, the differentially expressed genes analyzed in the first case study are taken from the authors supplementary file Table S4 [23] and are based on single cell sequencing data deposited at Gene Expression Omnibus GSE153947. Protein coding genes analyzed in the second case study [27] are acquired from ENSEMBL [2].

## Abbreviations

CGI: CpG Island
ChIP: Chromatin immunoprecipitation
DEG: Differentially expressed gene
lncRNA: Long non-coding ribonucleic acid
miRNA: Micro ribonucleic acid
OGRE: Overlapping annotated genomic regions
SHREC: Shiny interface for region comparison
SNP: Single nucleotide polymorphism
TSS: Transcription start site
