# Supplementary figures and images for: OGRE: calculate, visualize, and analyze overlap between genomic input regions and public annotations
Source: BMC Bioinformatics. 2023 Jul 26;24:300. doi: 10.1186/s12859-023-05422-w (PMC10369718; doi:10.1186/s12859-023-05422-w)

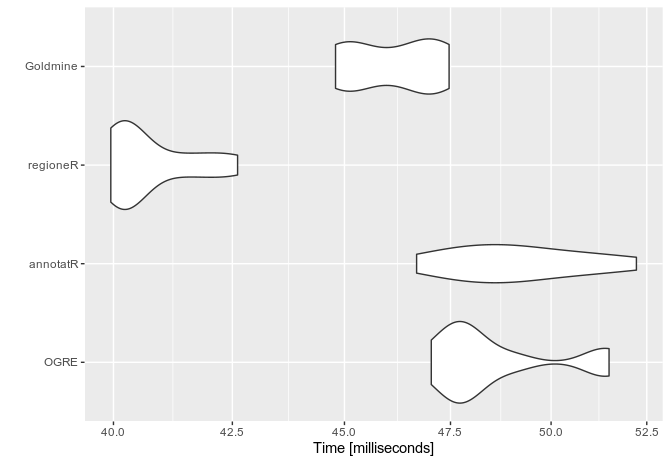

Supplement: Supplementary file 2 — Additional file 2: Figure S1. Computation times. Benchmark of overlap calculation by tools Goldmine, regioneR, annotatr and OGRE using two, 20,000 lines long input files with 10 runs each, computation time reported in milliseconds. [file 12859_2023_5422_MOESM2_ESM.png]

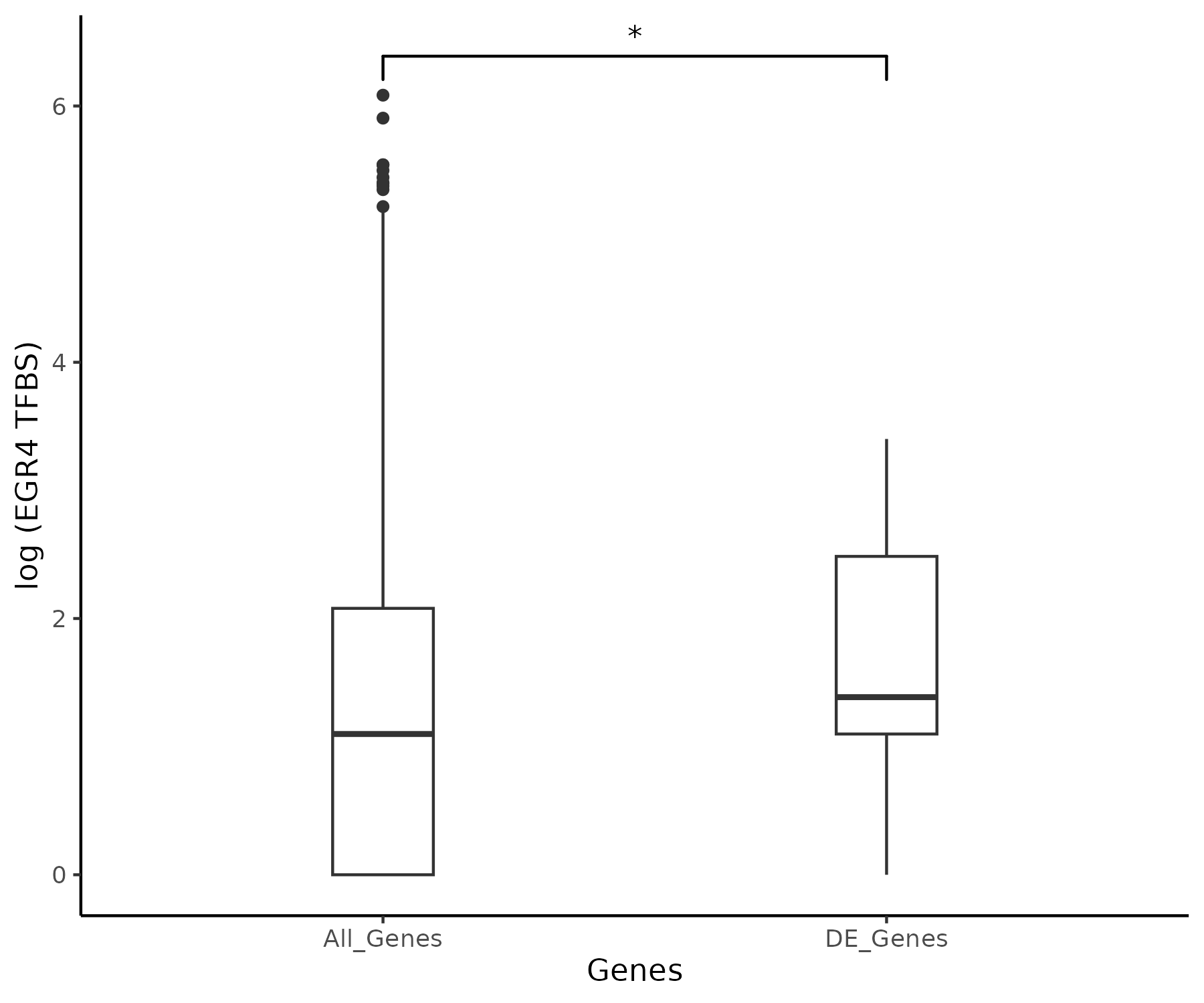

Supplement: Supplementary file 3 — Additional file 3: Figure S2. EGR4 TFBS. Number of EGR4 TFBS of all genes when comparing DEGs and GENCODE v30 release genes. [file 12859_2023_5422_MOESM3_ESM.png]
