# Supplementary material for: OGRE: calculate, visualize, and analyze overlap between genomic input regions and public annotations
Source: BMC Bioinformatics. 2023 Jul 26;24:300. doi: 10.1186/s12859-023-05422-w (PMC10369718; doi:10.1186/s12859-023-05422-w)
Supplement: Supplementary file 4 — Additional file 4: Table S1. Benchmark statistics. Benchmark statistics of overlap calculation by tools Goldmine, regioneR, annotatr, and OGRE showing detailed calculation times (min, lq, mean, median, uq, max). [file 12859_2023_5422_MOESM4_ESM.docx]

| expr | min | lq | mean | median | uq | max | neval | cld |
| --- | --- | --- | --- | --- | --- | --- | --- | --- |
| OGRE | 0.047036966 | 0.04762969 | 0.0485658514 | 0.0478694975 | 0.049048754 | 0.051500031 | 10 | c |
| annotatR | 0.046686393 | 0.047819224 | 0.0491320625 | 0.0490144555 | 0.050441128 | 0.052223479 | 10 | c |
| regioneR | 0.039945907 | 0.040127414 | 0.0407629375 | 0.0403351165 | 0.041350282 | 0.042613466 | 10 | a |
| Goldmine | 0.044795811 | 0.045100409 | 0.0461264511 | 0.046281313 | 0.047008053 | 0.047466006 | 10 | b |
